# Conducting a large, multi-site survey about patients’ views on broad consent: challenges and solutions

**DOI:** 10.1186/s12874-016-0263-7

**Published:** 2016-11-24

**Authors:** Maureen E. Smith, Saskia C. Sanderson, Kyle B. Brothers, Melanie F. Myers, Jennifer McCormick, Sharon Aufox, Martha J. Shrubsole, Nanibaá A. Garrison, Nathaniel D. Mercaldo, Jonathan S. Schildcrout, Ellen Wright Clayton, Armand H. Matheny Antommaria, Melissa Basford, Murray Brilliant, John J. Connolly, Stephanie M. Fullerton, Carol R. Horowitz, Gail P. Jarvik, Dave Kaufman, Terri Kitchner, Rongling Li, Evette J. Ludman, Catherine McCarty, Valerie McManus, Sarah Stallings, Janet L. Williams, Ingrid A. Holm

**Affiliations:** 1 Center for Genetic Medicine, Feinberg School of Medicine, Northwestern University, 645 N. Michigan Avenue, Chicago, IL 60611 USA; 2 Icahn School of Medicine at Mount Sinai, New York, NY USA; 3 University College London, London, UK; 4 University of Louisville School of Medicine, Louisville, KY USA; 5 Cincinnati Children’s Hospital Medical Center, Cincinnati, OH USA; 6 Penn State College of Medicine, Hershey, PA USA; 7 Vanderbilt University Medical Center and Vanderbilt University, Nashville, TN USA; 8 Seattle Children’s Research Institute, Seattle, WA USA; 9 Marshfield Clinic Research Foundation, Marshfield, WI USA; 10 The Children’s Hospital of Philadelphia, Philadelphia, PA USA; 11 University of Washington, Seattle, WA USA; 12 National Human Genome Research Institute, National Institutes of Health, Bethesda, MD USA; 13 Group Health Research Institute, Seattle, WA USA; 14 Essentia Institute of Rural Health, Duluth, MN USA; 15 Geisinger Health System, Danville, PA USA; 16 Boston Children’s Hospital, Harvard Medical School, Boston, MA USA

**Keywords:** Survey, Consent, Multi-site, Genomics, Institutional Review Board, Cognitive interviews, Pilot

## Abstract

**Background:**

As biobanks play an increasing role in the genomic research that will lead to precision medicine, input from diverse and large populations of patients in a variety of health care settings will be important in order to successfully carry out such studies. One important topic is participants’ views towards consent and data sharing, especially since the 2011 *Advanced Notice of Proposed Rulemaking (ANPRM),* and subsequently the 2015 *Notice of Proposed Rulemaking (*NPRM) were issued by the Department of Health and Human Services (HHS) and Office of Science and Technology Policy (OSTP). These notices required that participants consent to research uses of their de-identified tissue samples and most clinical data, and allowing such consent be obtained in a one-time, open-ended or “broad” fashion. Conducting a survey across multiple sites provides clear advantages to either a single site survey or using a large online database, and is a potentially powerful way of understanding the views of diverse populations on this topic.

**Methods:**

A workgroup of the Electronic Medical Records and Genomics (eMERGE) Network, a national consortium of 9 sites (13 separate institutions, 11 clinical centers) supported by the National Human Genome Research Institute (NHGRI) that combines DNA biorepositories with electronic medical record (EMR) systems for large-scale genetic research, conducted a survey to understand patients’ views on consent, sample and data sharing for future research, biobank governance, data protection, and return of research results.

**Results:**

Working across 9 sites to design and conduct a national survey presented challenges in organization, meeting human subjects guidelines at each institution, and survey development and implementation. The challenges were met through a committee structure to address each aspect of the project with representatives from all sites. Each committee’s output was integrated into the overall survey plan. A number of site-specific issues were successfully managed allowing the survey to be developed and implemented uniformly across 11 clinical centers.

**Conclusions:**

Conducting a survey across a number of institutions with different cultures and practices is a methodological and logistical challenge. With a clear infrastructure, collaborative attitudes, excellent lines of communication, and the right expertise, this can be accomplished successfully.

## Background

Surveys have been widely utilized to elicit perceptions about health research and perspectives on issues relevant to health research policy. Given the important ethical, legal, and social implications (ELSI) challenges raised by genomics research, hundreds of surveys have been conducted with the aim of informing the analysis of ethics and health policy challenges (see Garrison, et. al. [1] for a review). While these challenges are important on national and international scales, most of these surveys have been conducted at single sites or using large online databases of participants willing to participate in surveys. Both of these approaches have significant limitations, including lack of generalizability, sampling bias, and lack of population diversity.

Given variation in local populations and the potential influence of community relationships with local institutions, development and implementation of surveys across a variety of populations from multiple institutions helps address these limitations. However, few surveys relating to the ELSI challenges of genomic research and particular participant preferences for consent in genomic research have been designed and implemented in a collaborative fashion across multiple sites [2–5].

Technical and organizational challenges are likely responsible for the relative paucity of multi-institutional studies on these topics, including diverse cultures at different institutions; particularly medical centers and academic health care centers, varying interpretations of human subjects guidelines, and acceptable practices regarding data collection from potential participants. In addition, researchers have their own preferred approaches to survey development and data collection.

Online databases of potential survey respondents, such as GfK (http://www.knowledgenetworks.com/ganp/), a database of US households that are continuously surveyed through an online format, are important alternatives to multi-site surveys but are often limited to participants with online access. Another approach to obtaining an informative sample of participants would be to identify patients who receive care in health care settings where research is conducted, and then broadly survey patients at those institutions. In order to obtain a sample that includes participants with diverse experiences and characteristics, however, it would be necessary to survey patients across a diverse set of health care institutions. Surveying large numbers of patients from several institutions would enable comparisons across different types of care settings and patient backgrounds that might not be possible using GfK. In this case, conducting a survey across multiple sites provides clear advantages to either a single site survey, using a large online database, or commercially available sampling frames.

The eMERGE (Electronic Medical Records and Genomics) Network undertook such a challenge in 2013. The eMERGE Network was initiated by the National Human Genome Research Institute (NHGRI) in 2007 (RFA HG-07-005) to “develop, disseminate, and apply approaches to research that combine DNA biorepositories with electronic medical record (EMR) systems for large-scale, high-throughput genetic research” [6]. A focus of eMERGE II, initiated in 2011 (RFA-HG-10-009, RFA-HG-10-010, RFA-HG-11-022), was to explore the implementation of genomic medicine, how genetic variants could be incorporated into the EMR for clinical use, and the impact on clinical care and patients. The 9 eMERGE sites (which included 13 institutions, 11 of which are clinical centers) were: Northwestern University (NU), Vanderbilt University Medical Center (VU), Group Health (GHC) and University of Washington (UW), Mayo Clinic (Mayo), Geisinger Health Care (GHC), Icahn School of Medicine at Mount Sinai (ISMMS), Children’s Hospital of Philadelphia (CHOP), Boston Children’s Hospital (BCH) and Cincinnati Children’s Hospital Medical Center (CCHMC), and Marshfield Clinic (MC), Essentia Rural Health Institute (ERHI) and Penn State. The Coordinating Center (CC) was at VU.

Issues around Consent, Education, Regulation, and Consultation (CERC) were addressed by the eMERGE CERC workgroup [7]. The CERC workgroup’s efforts were aimed at understanding patients’ views and attitudes about participation in genomic research, particularly when that research relies on close linkages with EMRs. In 2013, the eMERGE network received a supplemental NIH grant aimed at understanding how the policy changes proposed in the 2011 *Advanced Notice of Proposed Rulemaking (ANPRM)* for revisions to the Common Rule released by the Department of Health and Human Services (HHS) and the Office of Science and Technology Policy (OSTP) would affect research participation at institutions, like those in the eMERGE Network, where genomic biorepository research takes place [8]. The ANPRM changes aimed to improve protections for human subjects while facilitating research and reducing burden on investigators. The subsequent Notice of Proposed Rulemaking (NPRM), issued in September 2015 [9], included many of the same policy proposals raised in the ANPRM. Among the NPRM proposals is the recommendation to require informed consent for research using de-identified tissue samples and most clinical data, and that such consent can be obtained in a one-time, open-ended or “broad” fashion. Broad consent can be defined as a process in which participants agree prospectively to have their samples, genomic data, and/or health information retained for use in any future research deemed appropriate by the relevant oversight body [1]. Moreover, the Genome Data Policy requires obtaining consent for data sharing as a condition for funding of most genomics research. These policies will greatly affect research using biobanks.

Although much has been written about the use of broad consent for biobanking and the future use of samples, empirical research suggests most patients do not favor broad consent over other consent options [1] and that factors such as privacy and distrust, which may affect willingness to participate, are greater for minority participants [1, 10–12]. However, less is known about sociodemographic factors; such as SES, education, and rural and non-rural living, which may affect willingness to participate in a biobank and allow one’s sample and data to be used for future research (see Garrison et al. for a full review [1]) Further, the Precision Medicine Initiative [13] magnifies the importance of informing and recruiting a large and representative cohort for current and future genomic research in ways that are acceptable to patients and the public.

A joint effort across the eMERGE Network, led by the CERC workgroup, provided an ideal way to address issues related to sharing of research data and broad consent and to obtain input from a diverse sample of participants. The highly collaborative eMERGE Network has a long-standing track record in productive research [7, 14–18]. The Network included a large population of biobank participants recruited across a diverse range of health care settings; including both urban and rural, adult and pediatric, academic teaching hospitals, an integrated health system and a member-governed coordinated care system. Thus a large survey across the 11 clinical centers that were part of the eMERGE Network was an ideal venue for eliciting the views of both eMERGE biobank participants and non-participants on issues around broad consent. The multi-disciplinary CERC workgroup had valuable expertise in addressing complex ELSI issues around genomic research. Within the context of the ANPRM and its potential impact on patient recruitment in biobank research, the eMERGE CERC workgroup set out to examine patients views on broad consent, governance and oversight of genomic research, and data sharing. Data sharing can be defined as making data available to other investigators though managed or open databases. A subcommittee of the CERC workgroup, the “CERC Survey workgroup”, was organized with at least one member from each eMERGE institution and the CC represented on the subcommittee.

In this paper we describe the collaborative effort among the members of the eMERGE Network CERC Survey workgroup to fill this important gap in knowledge by providing much-needed, large-scale quantitative data in the form of a survey sent to 90,000 patients and parents of patients across the 11 eMERGE clinical centers. The aims of this paper are to: 1) describe the process for developing and implementing the survey, 2) present the specific challenges of conducting a survey across diverse geographies and populations in a large network and the methods we developed to meet those challenges; including an organizational structure, separate but coordinated IRB approvals, and a survey development and implementation process that took into consideration the characteristics at each site.

## Methods

The CERC Survey workgroup first created an organizational structure to carry out the project. Co-chairs of the CERC workgroup, Maureen Smith (NU) and Ingrid Holm (BCH), led the effort and were responsible for facilitating communication, setting deadlines, disseminating information (with the CC), working with consultants and contractors (with the CC), communicating updates to the eMERGE steering committee, and reporting to the NHGRI/NIH. (The eMERGE steering committee is comprised of the principle investigators from each of the sites.) The workgroup divided the project into tasks and for each task established a committee to carry out the task. The committees and their leaders are listed in Table 1. The workgroup also engaged external individuals with expertise in cognitive interviewing methods, and in conducting biobank consent research, to augment the expertise of its members. The CERC Survey workgroup conducted weekly/biweekly conference calls throughout the entire study period (over 2 years), and each committee held conference calls weekly during periods of their most intense activity. The CERC Survey workgroup also met in person three times a year during eMERGE steering committee meetings.

**Table 1:** Committee name and leadership of each committee

Committee name	Committee leads
IRB Protocol	Jen McCormick and Sharon Aufox
Cognitive Interviews	Melanie Myers
Systematic Literature Review	Nanibaa’ Garrison
Survey Development	Saskia Sanderson
Sampling Strategies	Jonathan Schildcrout
Data Management	Kyle Brothers
Survey Analysis	Jonathan Schildcrout

### Systematic literature review

The Systematic Literature Review committee [Lead- Nanibaa’ Garrison (VU)] conducted a review of the literature to define gaps in current literature regarding patients’ acceptance of various forms of consent and factors associated with willingness to consent. The review focused on studies about broad consent and data sharing, biobank governance, data protection, and return of results. Database searches were conducted between October and December 2013 with an update in March 2015. The following databases were used: MEDLINE via the PubMed interface, Web of Science, National Reference Center for Bioethics Literature databases (EthxWeb, GenETHX), and Dissertation Abstracts International.

### Survey development

One of the most challenging aspects of the project was developing the survey itself, led by the Survey Development committee [Lead- Saskia Sanderson (ISMMS)]. The survey requirements included that it 1) address the key, complex issues of broad consent and data sharing, 2) be relatively short, 3) be written at an 8^th^ grade reading level, 4) be written in English as we cannot know *a priori* which potential participants only read languages other than English, and 5) be uniformly sent to 100,000 potential respondents (based on an estimate of 20% undeliverable rate, and an expected response rate of 20% for a total of 16,000 respondents,). Initial power calculations were based on having sufficient power to stratify analyses by subgroup. In addition, the CERC members were spread from Seattle to London (8 time zones), increasing the logistical challenges. 

### Population Sampling

The sampling strategy committee [Co leads- Jonathan Schildcrout (VU) and Nathaniel Mercaldo (VU)] designed the population sampling plan to be implemented across all 11 clinical centers. Our inclusion criteria included patients with a valid address (only one per household), and at least one inpatient or outpatient contact with a participating eMERGE clinical site between September 1, 2013 and August 31, 2014, for a population of approximately 2.4 million individuals. We used a recent one-year time horizon to minimize the chance that the patient had moved. Demographic variables (age, gender, race, and ethnicity) were obtained from the EMR at each institution even though such data may be not be entirely accurate and may have varying degrees of availability across sites. To bolster missing EMR data, and to ensure ascertainment of other key sociodemographic data, patient EMR data were linked to US census data files. The linkage process involved geocoding each patient’s home address (i.e., converting an address to a latitude and longitude value) and identifying the corresponding census block-group identifier using the geocoded address [19]. Geocoded address and Urban Area Criteria data were used to determine non-rural living while the census block-group identifier and the data from the American Community Survey were used to impute missing race, ethnicity and education [20]. The block-group level is the most granular level of data that the census provides, thus the block-group level was used to obtain the most accurate estimates as possible. While geocoding of patient addresses was done at most sites, addresses for those sites without the required expertise were geocoded at the CC, permitted by the eMERGE data sharing agreement. A stratified sampling plan was developed where sampling strata were defined using the combined EMR and Census-imputed sociodemographic variables.

### IRB protocol

There were two potential approaches to obtaining IRB approval. We could have a central IRB in which each institution cedes review to the central IRB as per a reliance agreement. Alternatively, we considered obtaining IRB approval from each of the 11 clinical centers. Since it was not clear that all sites would cede review to a central IRB, we decided to obtain IRB approval separately at each institution. As each IRB submission had to include an almost identical protocol and identical materials to be sent to potential participants, a single IRB protocol was developed by the IRB Protocol committee [Co leads- Jen McCormick (Mayo) and Sharon Aufox (NU)]. The committee developed introductory and reminder letters and postcards. Each site requested a waiver of informed consent due to minimal risk of the study. Completion and return of the questionnaire would assume passive consent. All study documents were submitted to each institution’s IRB in a coordinated manner.

### Cognitive interviews

Once a near-to-final version of the survey was developed, the Cognitive Interview committee [Lead- Melanie Myers (CCHMC)] was tasked with conducting cognitive interviews to determine whether the questions elicited the information they were designed to capture [21, 22]. The committee lead is an experienced qualitative investigator and she trained the interviewers at the participating sites. The cognitive interviews focused on the language, comprehensibility, decision processes, and relevance of each item in the survey (unpublished). We planned to conduct in person interviews with a convenience sample of up to 10 patients (or parents at pediatric sites) at each of 6 sites. Each cognitive interview was expected to take 60–90 min and participants would be compensated for their time. Sites for cognitive interviews were selected for non-white populations, individuals from rural areas, and having lower income.

### Piloting the survey

The survey was piloted to gather data on responses, including an evaluation of missed answers, problems with skip patterns, and straight-lining (selecting the same response for a series of questions without properly reading through each item). A total of 1500 individuals were selected for participation in the pilot survey. At each institution, a total of either 75 (2 institutions) or 150 (9 institutions) participants were identified by randomly selecting one from each sampling stratum. An initial survey pre-notice postcard was sent to each individual to receive the survey, letting them know the survey would be mailed to them shortly and providing them with contact information in case they had any questions. The survey mailing, which included an invitation letter, a non-contingent pre-incentive $2 bill, a scannable mail questionnaire, and a self-addressed business reply envelope, was mailed to participants one week later. The invitation letter provided both a unique identifying number (ID) and a simple URL, http://www.biobanksurvey.org/, to access the internet version of the survey. Respondents had the option to complete the survey on paper or online. Those who chose to complete the survey online were able to type the web address into a browser, enter their ID, and complete the survey through the REDCap survey interface [23].

### Survey implementation

In order to examine views of individuals recruited from multiple institutions across the US without confounding from between-site methodological differences, we planned to implement the survey uniformly across the institutions. We considered alternate methodologies for dissemination, including an email invitation with a link to a web-based survey. This would be less costly than a paper survey and would allow electronic capture of responses. To further ensure consistency with survey implementation, we decided to use an outside vendor for survey printing and mailing. The outside vendor required each site to send patient contact information (identifiable information) outside the institution to the vendor. All sites sought IRB approval for the vendor and business associates agreements (BAA), as needed.

## Results

### Systematic literature review

**Fig. 1:**
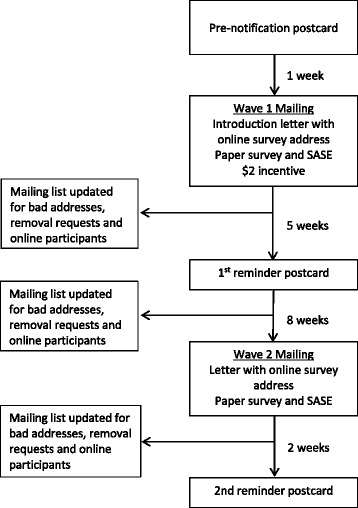
Survey mailing procedures with times between each step noted

The committee reviewed empirical studies carried out in the US after 1990 [1] and included studies using a variety of methodologies, including qualitative, quantitative, and mixed methods. Demographic characteristics of participants were reviewed to determine populations that may not have been included in previous studies. The literature review was a key component in our hypotheses generation to define our key survey questions and inform the development of the survey and our sampling methodology. Through the electronic database searches and manual review of articles and bibliographies, 3,205 relevant citations and abstracts were found (Fig. 1). After reviewing titles and abstracts, we excluded 2,714 studies that did not meet our criteria. We assessed the full text of the 491 remaining studies and excluded another 440 articles because they (i) did not address biobanking, consent, or data sharing (*n* = 403); (ii) were not conducted in the United States (*n* = 206); or (iii) were not obtainable (*n* = 1). Fifty-one publications comprising 48 unique cohorts met our inclusion criteria. We found that most studies about consent for biobank participation were small, and that individuals who were non-Caucasian, of lower income, and/or less educated were underrepresented. Additionally, the approach taken by most studies was to offer participants options about which consent model they preferred. The results of the systematic literature review informed both the survey content, design, and the targeted populations for this study.

### Survey development

Informed in part by the results of the literature review, the Survey Development committee first finalized the aims and hypotheses and then “operationalized” the aims by defining the terms and identifying domains of interest and variables within the aims. The committee developed a conceptual framework that contained both distal (participant level characteristics) and proximal (biobank level characteristics) variables potentially influencing our primary outcome. The committee collected surveys from previous studies, compiled a collection of questions from these surveys, and chose questions to modify for this survey. In order to manage this complex task, the committee divided the task into “domains” and organized domain subgroups within the committee, each of which identified survey questions within their domain to bring back to the group.

One key question that the workgroup needed to address was the structure of the survey. Based on our conceptual framework, an important aim of the study was to determine how proximal influencers, and in particular biobank-level characteristics – specifically consent approaches and data-sharing models – would affect willingness to participate in a biobank. However, previous research had suggested that survey respondents, when given a choice and asked to compare these types of biobank-level characteristics, may have difficulty considering practical trade-offs. [24, 25]. These previous studies have generally found that, when given the choice, patients tend to favor more restrictive consent and data-sharing approaches. However, this is not reflective of the real world. In practice, patients are not given a choice, but rather are invited to participate in a biorepository for which a consent and a data-sharing approach have already been selected. In order to simulate this real-world condition, and because we had a large population to survey, we decided to randomize respondents to one of three biobank scenarios with different consent and data sharing approaches, and to assess their willingness to participate in the scenario they were randomized to as the primary outcome (Table 2). Using this experimental rather than purely cross-sectional survey study design also gave us greater ability to manipulate the consent and data sharing features presented to participants, and conduct between-participants rather than within-participants comparisons. Once we made this decision, the committee had to identify the key constructs that were important to assess, drawing on the domains identified earlier. The constructs and domains were identified in an iterative process that included the findings from the systematic literature review as well as input from outside experts.

**Table 2:** The three consent and data sharing scenarios presented to survey participants

Consent Type	Data sharing	Biobank Description (All scenarios begin with: Please imagine that you have been asked to allow your health information to be placed in a biobank at your local hospital or healthcare organization. If you agree to take part in the biobank, the biobank will share your health information with researchers at your local hospital or healthcare organization who wish to use the health information in the biobank for research. In addition, this particular biobank will….)
Tiered Consent	Restricted Database	…share your health information with researchers at your local hospital or healthcare organization who wish to use the health information in the biobank for research. In addition, this particular biobank will also place your health information in large national databases. This is to make it easier for researchers across the world to do research with the health information. Researchers from other hospitals, healthcare organizations, companies, and government agencies concerned with health in the United States and in other countries can apply to use your health information. When you sign up, you will be asked what types of medical research you will allow your health information to be used for The biobank will remove your personal information such as name, address, social security number, and birth date that could identify you before it is shared.
Broad Consent	Restricted Database	…share your health information with researchers at your local hospital or healthcare organization who wish to use the health information in the biobank for research. In addition, this particular biobank will also place your health information in large national databases. This is to make it easier for researchers across the world to do research with the health information. Researchers from other hospitals, healthcare organizations, companies, and government agencies concerned with health in the United States and in other countries can apply to use your health information. When you sign up, you will be agreeing for your health information to be used for all kinds of medical research. The biobank will remove your personal information such as name, address, social security number, and birth date that could identify you before it is shared.
Broad Consent	Publicly Accessible Online Database	…share your health information with researchers at your local hospital or healthcare organization who wish to use the health information in the biobank for research. In addition, this particular biobank will also place your health information in a large online database that anyone in the public can access. This is to make it easier for researchers across the world to do research with the health information. When you sign up, you will be agreeing for your health information to be used for all kinds of medical research. The biobank will remove your personal information such as name, address, social security number, and birth date that could identify you before it is shared.

Another challenge was how to assess issues relating not only to adults’ participation in biobank research, but also issues relating to children’s participation. Three of the institutions were large pediatric medical centers (BCH, CCHMC, and CHOP), and one of the challenges was to determine how to obtain the views of parents without the need for a separate survey, which could have been prohibitively costly. Recognizing that many adults at the non-pediatric centers likely had children less than 18 years of age, we decided that all sites would use the same survey to elicit adults’ views on themselves and their youngest child less than 18 years of age.

A final challenge in survey development was to decide on how the final survey would be sent out. The decision was made to send the final survey package to the potential participants including a cover letter that introduced and explained the purpose of the study, and general information required to inform participants about the survey, their right not to participate, and who to contact should they have questions. The survey began with one page providing an explanation of the purpose of the survey, definitions of terms such as “health information” and “biobank,” and a detailed description of the purpose and structure of biobanks that was presented in a question and answer format. The survey scenario was presented and followed by the primary question about willingness to participate in the biobank. Following our conceptual framework, questions in the following domains were included in the survey: attitudes towards taking part in a biobank (perceived benefits, concerns, information needs), attitudes towards enrolling a child in a biobank (perceived benefits, concerns), trust in the healthcare system and medical researchers, privacy concerns, health-related items, and demographics.

### Population sampling

The Systematic Literature Review committee found that, while the relationships between gender (and to a lesser degree race) and views on consent and data sharing have been studied thoroughly, very little was known about the impact of socioeconomic status, education, rural residence, age, and ethnicity. Because the eMERGE network includes 11 large medical centers, we were well positioned to develop a study design that would enrich the sample with understudied populations and would therefore allow us to examine characteristics associated with views and attitudes that have not yet been well studied.

After combining EMR data with census data, and supplementing missing EMR data with census data, we developed sampling strata based on a cross classification age (> = 35 or <35 for adult sites, > = 12 or <12 for pediatric sites), gender, race (white, black, Asian, Native American, Pacific Islander, other race), ethnicity (Hispanic or not), education level of adults greater than 25 in the census block group (<12 years, 12- < 16 years, > = 16 years), and rural residence (yes or no). We then conducted a disproportionate stratified sampling design with the goals of oversampling racial and ethnic minorities, younger adults, those likely to have lower education, and those living in rural regions. Details of the sampling strategy will be made available in the publication of survey results.

### IRB protocol

The greatest regulatory challenge was that all changes to the materials, even if very minor, required all institutions to change the documents and send an amendment to their respective IRB since the materials had to be exactly the same at each site. All revisions were compiled by the IRB Protocol committee and a single final revision was submitted to all institutions’ IRBs. Although tedious, this approach proved successful, and final approvals were received from all institutions’ IRBs prior to conducting the survey. All institutions’ IRBs approved the waiver of informed consent.

Another challenge to local and full group IRB approval grew out of the strategy to use an outside vendor for survey printing and mailing. The outside vendor required each site to send patient contact information (identifiable information) outside the institution to the vendor. With these requirements having the possibility of creating delays and disagreement among the various IRBs, site investigators engaged in pre-emptive conversations with directors of their IRBs, IRB staff, and representatives from their institutions’ privacy office. This approach involved 9 of 11 sites signing a HIPAA Business Associate Agreement (BAA) with the vendor. One institution’s IRB could not agree to allow patient information to be sent outside the institution in the absence of patient consent. A strategy to comply with this local IRB requirement was developed for one site in which they would address and mail their surveys themselves. This required additional coordination in order for the mailings to be sent at the same time as the vendor mailings.

Timing between the pre-notification postcards and mailing the full survey was paramount to honoring opt-out requests. For the first mailing, however, the time between pre-notification postcards and full survey mailings was short and a number of individuals received surveys that had opted out. Most sites filed notification of these minor violations with their IRB and no further action was required. One site’s IRB required that the survey mailing needed to be stopped to allow time to remove opt-out requests from further survey mailings. This required all sites to stop mailings until the one IRB agreed to restart the process.

### Cognitive interviews

Forty cognitive interviews were conducted in person with a convenience sample of up to 10 patients (or parents at pediatric sites) at each of 6 sites. The survey was refined in an iterative process, with revised versions of the survey used in successive rounds of interviews. Results of the cognitive interviews led to simplifying language, additional explanations about biobanks, and rewording of questions about a child’s participation in a biobank.

### Survey data management

Working with an outside vendor for survey administration had benefits but also challenges. Each institution required a separate contract with the vendor and the contracting process at each of the 11 institutions varied in language, length of time to signed agreement, and in length of time to paid invoices that were required before work could proceed.

The vendor printed the postcards and letters with an institution-specific logo, signature, and contact phone numbers for individuals wishing to opt out of study mailings. This meant that each institution received and managed telephone contact with potential participants or their representatives who had questions about the study, wanted to opt-out of receiving mailings, or to report the potential participant’s participation was not possible (e.g. death). A central REDCap database was created by the REDCap Database committee [Lead- Kyle Brothers (CC)] to document all refusals and removals [23]. This committee also designed an internet survey that participants could access online using an URL and access code provided in the invitation letters.

All completed surveys were returned directly to the vendor who scanned and scored the survey forms. In addition, the undeliverable mailings sent from the vendor were also returned to the vendor. Periodic data files were sent by the vendor to the CC with information about undeliverable surveys, records for which a paper survey was received, and the survey responses.

### Piloting the survey

In considering the best manner to distribute the survey, we took into consideration that all sites would use the same methodology. Although email surveys would have been less costly, email addresses for patients were not available at all sites, and some sites did not find email surveys to be acceptable based on their interpretation of HIPAA guidelines. Additionally, not all potential participants had access to email, creating selection bias. Thus one major impact of having multiple sites was that it constrained our approach to survey implementation. Ultimately, we were able to use a mixed-mode design of mail and internet survey; however, for consistency, all survey pre-notifications, invitations, and reminders were sent by mail only.

For the pilot, 1500 surveys were mailed, 5% were undeliverable, with 2 opt outs. The response rate was 11% (there were no follow ups to the initial survey mailing). Analysis of pilot survey data found few missed answers, and no problems with straight-lining. While skip patterns were not an issue, some respondents who had children over the age of 18 answered the section meant for participants who had a child under the age of 18. We therefore added additional language to emphasize the section was only meant to be answered if the participant had a child under the age of 18. Based on the small number of opt out responses, we revised our number of full surveys to send out to 90,000 potential respondents.

### Final survey

The full survey was sent to 90,000 individuals at the 11 institutions following the same procedure used in the pilot (See Fig. 1). We followed procedures previously demonstrated to increase likelihood of response to a mailed survey [26, 27] including an announcement postcard, a small incentive ($2), a reminder postcard, a second mailing of the survey, and a second reminder postcard. Thirteen thousand surveys were returned and available for analysis for a 15.8% response rate. Survey results are described in detail in another publication. Between mailings, potential participants could call a local telephone number to opt-out of receiving further mailings. Opt-out information was reported by each institution through a central REDCap survey to the CC. The opt-outs were collated prior to each mailing to remove names from the list. Due to printing deadlines, it was not always possible to remove individuals who opted out from the next mailing. To address this possible issue, individuals who opted out were advised that they should ignore and discard any subsequent mailing from the study. As noted in the IRB Protocol section, an error in the mailing list for one of the mailings resulted in mailings to some individuals who had previously opted out.

## Discussion

Policy changes, such as those proposed in the ANPRM in 2011 and the NPRM in 2015, by HHS and OSTP, and the NIH Genome Data Sharing Policy, have potentially significant implications for institutions all across the US conducting biobank research, and for the patients enrolling in such research. The changes include the recommendation to require informed consent for research using de-identified tissue and samples and most clinical data, and that such consent be obtained in a one-time, open-ended or “broad” fashion. The only way to study patients’ views of these policies is to ask them directly, and one way to do so is through a survey targeting patients most likely to be impacted by these changes. The eMERGE Network provided an excellent framework to address these issues as it included a large population of patients and biobank participants in a diverse range of health care settings across the US.

There were several key components that led to our success in developing and implementing a large survey across multiple institutions. The first is that the CERC workgroup was well established and had a long history of working together on ELSI-related projects through the eMERGE Network. In addition, the workgroup also had a history of working both as a full team and with specialized subgroups to explore specific focus areas within the CERC venue. Thus, the group was well equipped to work as a team on this project.

Another key reason the group was successful was that from the start of the project the CERC Survey workgroup identified key tasks and organized into multi-disciplinary committees for each task. The CERC workgroup included individuals with a variety of skill sets who enthusiastically committed to this new project. Support from the CC was critical to the success as the CC had a long history of managing all aspects of the eMERGE Network and had experience managing people, timelines, and deliverables. Thus the co-chairs and the CC kept the CERC Survey workgroup on task and accountable at each step in the process.

There were also clearly a number of challenges. Obtaining IRB approval at all sites was time consuming. We decided to obtain IRB approval from each of the institutions instead of having a central IRB. There were several reasons for this decision. After conversations with their various IRBs, some of the sites were concerned that there would be issues with ceding review to a central IRB, especially those with limited experience with reliance agreements. We also felt that obtaining IRB approval at each institution separately would be quicker. Because this was a minimal risk study of adults, it could undergo expedited review instead of full IRB committee review. The biggest challenge was the coordination of the submission and requirements of individual institutions. Again, having a committee dedicated to the IRB submission facilitated the coordination of the IRB approvals in a timely manner.

Not only did the IRB protocol require approval at each site, there were other tasks that individuals outside of the eMERGE CERC workgroup had to undertake. The major tasks in this category were obtaining BAAs at 9 of the 11 clinical centers, and finding individuals at each site who could geocode the sample and transfer the data to the CC. All institutions had the staff and experience to obtain and execute BAAs. Geocoding was more of a challenge as not all institutions had expertise in this area and it involved working with patient data and large files that needed to be transferred across sites. In addition, through geocoding, we intentionally sent surveys to potential participants who, based on demographic characteristics, are less likely to participate in research, further limiting our sample.

One challenge was the development of the survey itself. The large and multi-disciplinary CERC Survey workgroup provided considerable expertise, but also at times threatened to become unwieldy. Similarly to how the entire project was organized into tasks, the Survey Development committee was divided into five sub-subgroups that focused on each of the domains in our conceptual model. The Survey Development leader also led the structure of the survey development into first defining hypotheses, then domains, and then survey questions. Organizing the tasks and the Survey Development committee itself were key to the success of the project.

Finally, another decision that was key to the success of the survey’s implementation was to use an outside company to implement the survey. Although 1 site was unable to use the vendor for mailing, the other 10 were. Overall, the use of the vendor greatly facilitated the implementation of the survey. A minor issue was people mistakenly receiving the survey who had opted out after the first notification postcard.

We found that some of our assumptions were incorrect, including the undeliverable rate. We found the nondeliverable rate to be lower than anticipated in the pilot, and therefore decreased the total number of surveys mailed to 90,000 instead of the original 100,000. This ended up reducing estimated costs for mailing the survey. Additionally, choosing to use and coordinate with 11 different IRBs led to some delays in our study, due in large part to the different methods for managing the missed opt-outs at each institution but also to the different language requirements for approved documents. Acceptance of a single IRB might have reduced time but not allowed for local issues to be managed effectively.

## Future studies

As NIH has now adopted a policy for use of a central IRB for multisite human subjects research (Notice Number: NOT-OD-16-094), this will impact future studies such as ours. Further research into the use of a central IRB and it’s effects on issues such as maintaining local culture, increasing efficiencies, and the use of outside vendors for managing participant data will help to define the benefits of a central IRB. In addition, while we made efforts to increase diversity of participants in our study by stratifying our participant population prior to mailing surveys, we recognize that this method may not have been ideal. Also, because our survey was in English only, we likely decreased responses from non-English speaking participants. Further research into methods to increase survey participant diversity through methods other than linking with census data would further contribute to knowledge about study design.

## Conclusion

Conducting a survey across a number of institutions with different cultures and practices is a methodological and logistical challenge. However, with a clear infrastructure, collaborative attitudes, excellent lines of communication, and the right expertise, this can be done with much success.

## Abbreviations

*ANPRM*: *Advanced Notice of Proposed Rulemaking*
NPRM: Notice of Proposed Rulemaking
HHS: Health and Human Services
OSTP: Office of Science and Technology Policy
eMERGE: Electronic Medical Records and Genomics
NHGRI: National Human Genome Research Institute
EMR: Electronic medical record
ELSI: Ethical, legal, and social implications
NU: Northwestern University
VU: Vanderbilt University Medical Center
GHC: Group Health
UW: University of Washington
Mayo: Mayo Clinic
GHC: Geisinger Health Care
ISMMS: Icahn School of Medicine at Mount Sinai
CHOP: Children’s Hospital of Philadelphia
BCH: Boston Children’s Hospital
CCHMC: Cincinnati Children’s Hospital Medical Center
MC: Marshfield Clinic
ERHI: Essentia Rural Health Institute
CC: Coordinating Center
CERC: Consent, Education, Regulation, and Consultation
IRB: Institutional Review Board
HIPAA: Health Insurance Portability and Accountability Act
BAA: Business Associate Agreement
NIH: National Institutes of Health
